# Polyphenols Targeting MAPK Mediated Oxidative Stress and Inflammation in Rheumatoid Arthritis

**DOI:** 10.3390/molecules26216570

**Published:** 2021-10-30

**Authors:** Tapan Behl, Tanuj Upadhyay, Sukhbir Singh, Sridevi Chigurupati, Amal M. Alsubayiel, Vasudevan Mani, Celia Vargas-De-La-Cruz, Diana Uivarosan, Cristiana Bustea, Cristian Sava, Manuela Stoicescu, Andrei-Flavius Radu, Simona Gabriela Bungau

**Affiliations:** 1 Department of Pharmacology, Chitkara College of Pharmacy, Chitkara University, Rajpura 140401, Punjab, India; sukhbir.singh@chitkara.edu.in; 2 Amity Institute of Pharmacy, Amity University Gwalior, Gwalior 474005, Madhya Pradesh, India; tanujupadhyay567@gmail.com; 3 Department of Medicinal Chemistry and Pharmacognosy, College of Pharmacy, Qassim University, Buraidah 52571, Saudi Arabia; sridevi.phd@gmail.com; 4 Department of Pharmaceutics, College of Pharmacy, Qassim University, Buraidah 52571, Saudi Arabia; asbiel@qu.edu.sa; 5 Department of Pharmacology and Toxicology, College of Pharmacy, Qassim University, Buraidah 52571, Saudi Arabia; vasumpharmacol@gmail.com; 6 Faculty of Pharmacy and Biochemistry, Academic Department of Pharmacology, Bromatology and Toxicology, Centro Latinoamericano de Enseñanza e Investigación en Bacteriología Alimentaria, Universidad Nacional Mayor de San Marcos, Lima 15001, Peru; cvargasd@unmsm.edu.pe; 7 E-Health Research Center, Universidad de Ciencias y Humanidades, Lima 15001, Peru; 8 Department of Preclinical Disciplines, Faculty of Medicine and Pharmacy, University of Oradea, 410073 Oradea, Romania; diana.uivarosan@gmail.com (D.U.); cristianabustea@yahoo.com (C.B.); 9 Department of Medical Disciplines, Faculty of Medicine and Pharmacy, University of Oradea, 410073 Oradea, Romania; cristian.sava2004@gmail.com (C.S.); manuela_stoicescu@yahoo.com (M.S.); 10 Faculty of Medicine and Pharmacy, Doctoral School of Biological and Biomedical Sciences, University of Oradea, 410073 Oradea, Romania; andreiflavius.radu@gmail.com; 11 Department of Pharmacy, Faculty of Medicine and Pharmacy, University of Oradea, 410028 Oradea, Romania

**Keywords:** rheumatoid arthritis, TLR/MAPK, flavonoids, stilbenes, interleukin, TNF, oxidative

## Abstract

Rheumatoid arthritis (RA) is a chronic, systemic, autoimmune disorder, predominantly symmetric, which causes joint inflammation, cartilage degeneration and bone erosion, resulting in deformity and the loss of physical function. Although the management of RA has steadily improved, the pathophysiological mechanism is incompletely elucidated, and therapeutic options are still limited. Due to shortcomings in the efficacy or safety profiles of conventional RA therapies, therapeutic alternatives have been considered. Therefore, natural extracts containing polyphenolic compounds can become promising adjuvant agents for RA global management, due to their antioxidant, anti-inflammatory and apoptotic properties. Polyphenols can regulate intracellular signaling pathways in RA and can generate different immune responses through some key factors (i.e., MAPK, interleukins (ILs 1 and 6), tumor necrosis factor (TNF), nuclear factor light k chain promoter of activated receptor (NF-κB), and c-Jun N-terminal kinases (JNK)). The critical function of the Toll like-receptor (TLR)-dependent mitogen-activating protein kinase (MAPK) signaling pathway in mediating the pathogenic characteristics of RA has been briefly discussed. Oxidative stress can trigger a change in transcription factors, which leads to the different expression of some genes involved in the inflammatory process. This review aims to provide a comprehensive perspective on the efficacy of polyphenols in mitigating RA by inhibiting signaling pathways, suggesting future research perspectives in order to validate their use.

## 1. Introduction

Rheumatoid arthritis (RA) is a long-term, autoimmune, and inflammatory disease that mainly affects the synovial joints, which prompts bone and cartilage damage as the RA progresses [1]. Antibodies (such as anti-citrullinated protein antibodies (ACPA) and rheumatoid factor (RF)) have been detected in many patients with RA. It reduces patients’ functional capacity while increasing mortality and morbidity ratios [2]. Women are more affected than men. The dominance rate is 1% of the worldwide population. As of 2015, it is estimated that RA affects about 24.5 million people. This number includes 0.5 to 1% of adults in the developed world, with 5 to 50 per 100,000 patients newly added each year [3,4]. The disease etiology and pathogenesis are still unknown. Interactions between many factors, including hereditary and natural aspects, cause an incorrect adjustment of the immune response and an inflamed process that damages the synovial membrane.

Several explanations have been proposed in the absence of a complete understanding of the pathophysiological mechanisms underlying RA. Immunological disorders have been shown to occur several years before the appearance of signs and symptoms, a period of time known as the pre-RA phase [5].

The interactions between major genetic factors (protein tyrosine phosphatase non-receptor type 22, interleukin-6 receptor, tumor necrosis factor receptor-associated factor-1, signal transducer and activator of transcription 4, peptidylarginine deiminase 4, CC chemokine ligand 21, DNA methylation changes, Fc gamma receptor, major histocompatibility complex regions encoding human leukocyte antigen (HLA) proteins) and environmental factors (air pollution, occupational dust, smoking, gut microbiota, unbalanced diet, etc.) can lead to modified self-antigens by a process called citrullination [6]. Furthermore, the immune system can no longer recognize citrullinated proteins as self-structures. Antigen-presenting cells are stimulated to generate an immune response and carry the modified self-antigens into the lymph node. At this level, the activation of T cells occurs, which will lead to the activation of B cells by costimulation. Following some processes of hypermutation and class-switch recombination, B cells begin to proliferate and differentiate into plasma cells that generate autoantibodies (RF, ACPA, etc.), depending on the cellular precursors [5]. RF and ACPA are proteins produced by an immune system that has lost the ability to distinguish between self- and non-self-structures, so that in this case the tissues and organs can become targets by accident [7].

The activation of RA symptoms is incompletely elucidated, but the immunological processes can occur both in the synovium and in the synovial fluid. One of the best described mechanisms in the synovium is the cytokine release (IL-1, IL-6, TNF-α) from macrophages and plasma cells that can lead to the stimulation of osteoclast activity and production of matrix metalloproteinase (MMP), processes that can cause bone erosion and cartilage damage. Furthermore, neutrophils and immune complexes present in the synovial fluid are also responsible for cartilage and bone destruction by the action of MMP, complement system and reactive oxygen species (ROS) [5,8]. ROS were considered the main participant in this process [9].

The most common type of radical produced by living systems is ROS. The superoxide radical (O_2_), peroxyl radical (ROO), per hydroxyl radical (HO_2_), and hydroxyl radical (OH) are oxygen-derived radicals, as well as non-free radical species such as hydrogen peroxide (H_2_O_2_) and singlet oxygen (O_2_). The three most significant reactive nitrogen species (RNS) are nitric oxide (NO), nitrogen dioxide (NO_2_), and peroxy-nitrite (OONO) [10]. Atoms and elements with one or more unpaired electrons in the furthest orbital shell are known as free radicals [11].

They are unsteady, profoundly responsive, and last for a limited time. Free radicals can grab electrons from different mixtures to acquire dependability; as a reaction, the designated atoms lose their electrons and become free radicals, causing a chain response. ROS are fundamental to keeping up with cells’ redox conditions and engage with cell flagging, separation, expansion, development, demise, cytoskeletal control, and phagocytosis. Nonetheless, if ROS fixations transcend solid levels, they can harm cell segments such as fatty acids and phospholipids in the cell membrane (chains of amino acids and nucleic acids). On the off chance that a specific condition causes an unevenness among oxidants and antioxidants, preferring oxidants, redox flagging is disturbed, bringing about changes as well as sub-atomic damage. This cellular state known as oxidative stress can be caused by an overabundance of oxidants, a lack of antioxidants, or a combination of the two [12].

Antioxidants prevent the harmful effects of free radicals. Antioxidants are any molecules capable of scavenging free radicals or hindering the oxidation interaction within cells [13]. Superoxide dismutase- (SOD), catalase- (CAT), and glutathione (GSH)-related compounds are involved in the enzymatic disease control of cancer-suppressing reactions, glutathione peroxidase (GPx), glutathione reductase (GR), and thioredoxin reductase (TR). The most fundamental safeguard for non-enzymatic cell antioxidant responses is carotene, which is also required to treat the disease, or preventive minerals (cupper, ferritin, zinc, manganese, and selenium), as well as l-glutamyl-cysteinyl glycine [14].

One of the disorders that causes oxidative stress is RA. A five-fold expansion in the powerhouse of cell ROS induced in patients’ whole blood and monocytes, contrasted with healthy controls, shows that oxidative pressure is a pathogenic element of the sickness. As free radicals play an important role as secondary messengers in stimulation and the immunological cell response, they are indirectly involved in joint destruction [15]. T cells exposed to an extremely elevated level of oxidative stress become resistant to various signals, including those that control development and mortality, which may help maintain the unbalanced immune response. Simultaneously, free radicals directly affect joint cartilage by targeting its proteoglycan and by reducing and suppressing its synthesis [16].

In RA, oxidative harm to hyaluronic corrosive and lipoperoxidation outcomes, the oxidation of low-thickness lipoprotein, and carbonyl expansion brought about by protein oxidants, as well as DNA damage, have all been reported. ROS-actuated genotoxic events have additionally been associated with the transformation of p53 in RA-inferred fibroblast-like synoviocytes [17]. Additionally, cell reinforcement systems, if enzymatic, have been suggested to be compromised in RA. Reduced GR and SOD movement, as well as low GSH tocopherols, beta-carotenes, and retinol levels, were all connected [18].

The enhanced intra-articular pressure factor in RA joints is thought to be the cause of persistent oxidative pressure in the RA synovial membrane since it increases ROS generation in cell oxidative phosphorylation and creates continuous hypoxia/reoxygenation cycles. Hypoxia is a phenomenon found in RA joints that has been attributed to the inflammation reaction’s quick cell multiplication; in any case, considering the literature data, hypoxia happens before irritation, essentially in an animal arthritic model [19]. This series of symptoms occurs in human illness, as per the “Risk Model”, in which the synoviocyte is a damaged cell [20]. During an oxidative burst, enacted by phagocytic cells, oxidative stress can also increase. Smoking, narcotics, and UV light may all influence the disease. Different oxidant or cancer prevention agent pointers have been utilized to explore the association between oxidative stress and RA. Fatty acids, phospholipids, chains of amino acids, genomic alteration, and oxidation markers, as well as steps of enzyme action, cancer prevention agents, and direct predictions of free radicals, are examples of biomarkers [21].

Polyphenols are natural extracts, found mainly in specific parts of plants (fruits, roots, leaves), with well-known examples being apples, berries, citrus fruit, broccoli, cocoa, tea, and coffee. These plant-based compounds have various biological activities [22], with the chemical structure of these compounds obviously implying their activities/actions, both in vitro and in vivo [23]. Moreover, by assessing the biological activity of these natural polyphenolic compounds, beneficial effects have been demonstrated in the prevention and treatment of age-related disorders, skin damage, infections, malignancies and cardiovascular diseases, but their possible use in the management of RA is given by their antioxidant and anti-inflammatory activities. The antioxidant activity of polyphenols has been extensively studied, including free radical scavenging, the decrease in hydroperoxide production and the suppression of lipid oxidation [24].

A randomized cross-over study evaluated the antioxidant capacity of green teas at different polyphenol concentrations and showed linear correlations between the antioxidant contents of green teas and the antioxidant capacity of plasma [25].

Technological and medical advances have provided a better understanding of the interactions of various polyphenols with the pathways of inflammatory response. Polyphenols have anti-inflammatory properties due to several mechanisms, as follows:

- Regulation of cyclooxygenase-2 activity;
- Inhibition of eicosanoid-generating enzymes (phospholipase A2 and cyclooxygenase);
- Inhibition of NO release;
- Regulation of cytokines;
- Inhibition of NF-κB;
- Regulation of MAPK pathway [24].

Polyphenols have been basically subdivided into four well-known categories, as follows: phenolic acids, flavonoids, stilbenes, and lignans.

The current study aimed to evaluate the natural extracts of flavonoids, phenolic acids, stilbenes, and other phenolic compounds that have been studied for their anti-inflammatory and antioxidant properties against RA. Oxidative stress and inflammation in synovial joint tissues are associated with the progression and severity of this disease, also demonstrated by animal models with osteoarthritis (OA). The present literature indicates that polyphenols (such as quercetin, rutin, morin, etc.) show modulatory effects on the cells involved in inflammation, revealing their potential use in optimizing RA treatment management.

## 2. Pathogenesis of Rheumatoid Arthritis

Numerous investigations have shown the role of ROS in the progression of disease inflammation in long-term arthropathies such as RA [9]. As a result, gaining a better knowledge of the complicated interconnections across these pathways could aid in the development of new RA therapy and medication paths.

RA produces ROS through two main mechanisms: active polymorphonuclear cells (PMNs) and cell-necrosis in an inflammatory joint. Lipid peroxidation occurs if these reactive species are not scavenged. Polyunsaturated and unsaturated fats were oxidized during lipid peroxidation to shape lipid peroxyl revolutionaries, which then, at that point, led to the extra oxidation of polyunsaturated and unsaturated fats, possibly causing damage to the cell membrane. Lipoperoxidation products have been shown to cause oxidative damage in RA synovial fluids and tissue. In the plasma of RA patients were discovered impressively more prominent amounts of superoxide anions revolutionaries and H_2_O_2_. Extended SOD activity is likely to determine superoxide anion revolutionaries in plasma to produce hydrogen peroxide. Moreover, the CAT or glutathione detoxification of H_2_O_2_ was not found [26]. Increased blood lipid peroxidation in people might have been achieved by hydrogen peroxide being transformed to hydroxyl by iron due to decreased transferrin levels. Under normal conditions, nitric oxide (NO) is demonstrated to modulate T cell activities, while excessive NO generation might include T lymphocyte malfunction [27,28]. The plasma NO levels in RA patients were substantially different compared to the controls in the study. As with NO_2_, there is a solid negative link with GSH, which could be expected to compensate for the impacts of intracellular non-enzymatic antioxidative cycles due to their response to increasing NO_2_ generation [29].

A few examinations in RA patients have discovered signs of increased endogenous NO synthesis, proposing that NO overproduction may assume a part in the pathogenesis of the illness. The main site of NO in RA is the inflamed joint [21]. Several researchers found an association between serum nitrite content and RA sickness activity or radiographic damage, while others did not. In people with RA, a connection between disease action and the presence of oxidative stress was highlighted [30]. Different analysts have found no considerable links between disease action and the presence of oxidative pressure in RA patients. To shield the organic system from oxidative harm, many defense mechanisms have arisen. The connection between erythrocyte SOD and RA is not fully known [21].

## 3. Polyphenols and Rheumatoid Arthritis

Polyphenols act on three pathways to slow down the movement of RA: the inflammation, oxidative, and apoptotic pathways. Polyphenols fundamentally impact the inflammation system through the MAPK track and the guidelines of the NFATC1 quality in osteoblasts. MAPK, ILs 1 and 6, TNF-α, NF-κB, JNK, extracellular signal-directed kinase (ERK1/2), activator protein-1 (AP-1), and COX-2 represent a portion of the significant particles associated with these processes [31].

### 3.1. Phenolic Acids

The characteristic phenolic acids are hydroxybenzoic and hydroxycinnamic acids. Phenolic acids account for nearly 33% of the polyphenolic substances in our diet and can be found in all-natural plant substances; however, they are abundant in poisonous natural products. Normal phenolic acids incorporate caffeic corrosive, gallic corrosive, and corrosive. Phenolic acids have aggressive action on RA. When rodent monocytes and macrophage cells are pre-exposed for twenty-four hours to ferulic corrosive, which was discovered in grain and vegetable, natural products, and nuts, they influence the atomic characteristics of activated T cell C1 (NFATc1), c-Fos, NF-κB, Tartrate-safe corrosive phosphatase, network matrix metalloproteinases (MMP)-9, and cathepsins [32]. In arthritic rats’ liver and spleen cells were N-feruloylserotonin (N-f-5HT), a natural polyphenol derived from *Leuzea carthamoides*, inhibited C-reactive proteins (CRP), 12/15-lipoxygenases (LOX), TNF-α, empirical NO synthase (iNOS), and IL-1. The study employed 3 mg/kg of N-f-5HT and lasted 28 days [33]. Chlorogenic acid from *Gardenia jasminoides* inhibited p38, extracellular signals-directed kinase (ERK), and phosphorylation, and initiated the T cell yield of mRNA characteristics (NFATcl). Likewise, for 4 days, when ten, twenty-five, or fifty g/mM of CGA was provided to bone marrow macrophages (BMMs), lipopolysaccharide-induced (LPS) bone disintegration was supported in vivo [34].

TNF-α, IL-1 and IL-6 are pro-inflammatory cytokines involved in the control of the immune response in RA and are linked to inflammatory processes and the stimulation of osteoclast activity. Mitogen-activated protein kinases (MAPK) have a pivotal role in regulating the production of these pro-inflammatory cytokines, leading to joint inflammation and destruction [35]. Due to their involvement in various pathophysiological mechanisms, they have become potential therapeutic targets for the treatment of RA. TNF-α (etanercept, infliximab, golimumab, adalimumab, certolizumab pegol), IL-1 (anakinra, canakinumab, gevokizumab) and IL-6 inhibitors (tocilizumab, sarilumab, olokizumab) are biological drugs available on the pharmaceutical market for the treatment of RA. Furthermore, p38 MAPK is a promising target for many therapeutic agents that are in the second phase of testing [36].

**Figure 1:**
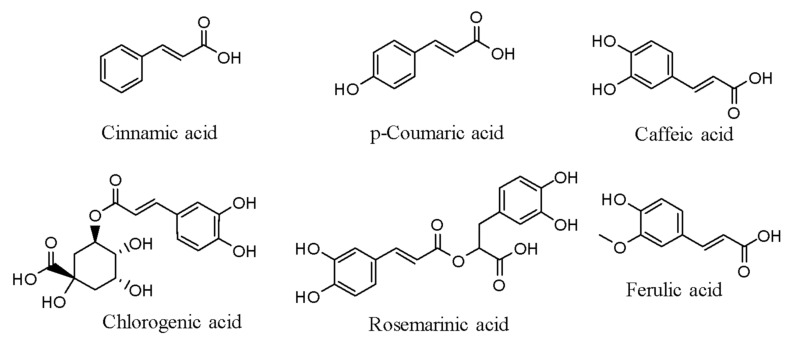
Chemical structures of a few phenolic acids.

The receptor activator of nuclear factors kappa-B-ligand (RANKL) and Thrombin receptor-activating peptide (TRAP) receptors support the inflammatory cytokines IL-1b, IL-6, IL-17, and the iNOS (COX-2) stimulating the synthesis of compounds and NF-κB-p65, p-NF-κB-p65, NFATc-1, c-Fos, and NF-κB-p65, and NF-κB-NF-κB-p65 [37]. The chemical structures of a few phenolic acids are presented in Figure 1.

### 3.2. Stilbenes

The stilbenes, 1,2-diphenylethylene, are subdivided into two types: The trans isomers are (*E*)-stilbenes, while the cis isomers are (*Z*)-stilbenes [38]. Stilbene is a polyphenol that has anti-inflammatory, cell survival, and antioxidant properties. The most notable of the above 400 natural stilbenes is resveratrol (RSV). RSV has recently been identified as a new possible therapeutic option for suppressing inflammation in a mouse model of collagen-induced arthritis. Moreover, starting from these results, clinical trials have been developed in order to demonstrate the beneficial effects of RSV on RA patients.

A randomized controlled clinical trial involving 100 RA patients has shown that adding RSV as an adjuvant to the conventional antirheumatic drugs (leflunomide, hydroxychloroquine, sulfasalazine, methotrexate) significantly improves the values of clinical (28 joint counts) and biochemical markers (C-reactive protein, TNF-α, erythrocyte sedimentation rate, IL-6), as well as the disease activity score [39]. The possible mechanism of action of RSV consists in the inhibition of MAPK signaling pathways by reducing ROS accumulation, along with alleviation of hypoxia-inducible factor 1α (HIF-1)-mediated angiogenesis [40].

Fibroblast-like synoviocytes (FLSs) are specialized cells located in the synovium. In the context of RA, FLSs are activated and can produce MMP, but can also stimulate RANKL expression, leading to bone erosions and cartilage destructions. The important role of FLSs in the pathogenesis of RA and their interactions with other cells suggests that these cell types could be a novel target for RA treatment [41].

Glycolytic inhibitors not only reduce the aggressive FLS phenotype but also prevent tissue and cartilage damage in several models of arthritis. The substance suppressed Beclin one, LC3A/B, and manganese-subordinated superoxide dismutase (MnSOD), and encouraged MtROSs in FLSs of reactive amyloids (AA) that were administered at doses of five, fifteen, and forty-five mg/kg of RSV over two weeks [42].

Akt, p38 MAPK, ERK1/2, COX-2, prostaglandin E2 (PGE2), nicotinamide adenine dinucleotide phosphate (NADPH) oxidases (ROS) [43], and NF-κB were all suppressed in FLSs in humans after a dosage of 50 g for 24 h. In a test using resveratrol at doses of 6.25, 12.5, 25, and 50 µM on a human synovial membrane, resveratrol had the same effect through modulating IL-1, MMP-3, p-Akt, and PI3K-Akt [39]. Literature data show that a three-month randomized controlled clinical experiment was conducted in which fifty patients were administered 1 g RSV capsules. RSV treatment had a considerable therapeutic benefit in RA, according to this study [39]. Swollen 28-joint count (SJC-28), Tender 28-joint count (TJC-28), CRP, erythrocyte sedimentation rate (ESR), uncarboxylated osteocalcin (UCOC), MMP-3, TNF, IL-6, and DAS28-ESR (Diseases Activities Score-28 for Rheumatoid Arthritis with ESR) levels are also decreased [44].

**Figure 2:**
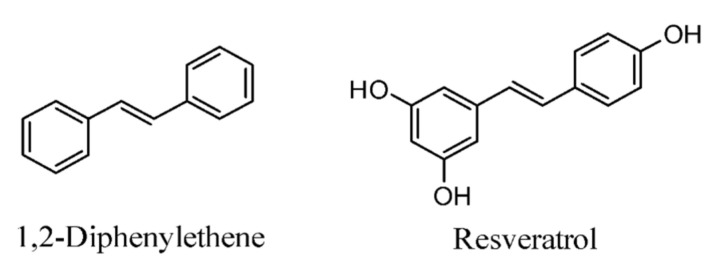
Chemical structures of stilbenes.

Moreover, at a 20 mg/kg dose, RSV eased RA indications by downregulating immunoglobulins G (IgG1, IgG2a). The outflows of IL-17 and interferon (IFN)-α were reduced after treating rat draining lymph node (DLN) cells and Th cells with forty M RSV for 3 days. Infusions of 30 M or 50 M for 3 days stifled TH-17 and IL-17 in a similar cell line. The chemical structures of 1,2-diphenyletene and resveratrol are presented in Figure 2.

### 3.3. Flavonoids

**Figure 3:**
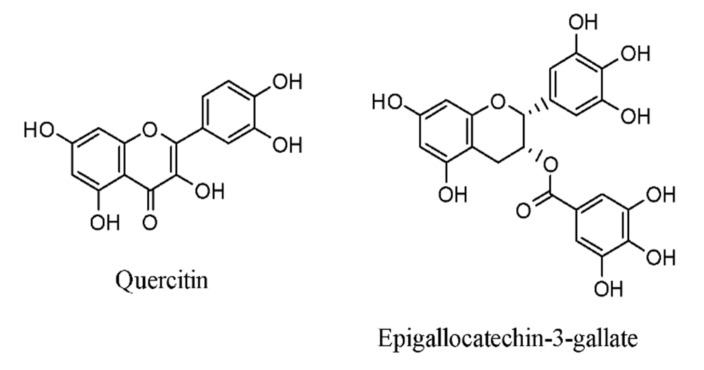
Structural representation of flavonoids.

Flavonoids are a kind of polyphenol composed of two phenyl rings organized into 15-carbon structures. Probably the most notable flavonoids are quercetin and epigallocatechin-3-gallate (EGCG), a flavonoid found in tea. The structures of quercetin and epigallocatechin-3-gallate are represented in Figure 3.

These natural compounds have anti-inflammatory properties and are hostile to cholinesterase and are therefore used to treat a variety of disorders. A flavonoid-rich diet, for example, has been linked to a lower risk of cardiovascular disease [45]. Citrus flavonoids can influence lipid metabolism and can be used to treat metabolic disorders. Flavonoids’ anti-inflammatory properties are used to alleviate the characteristics of RA [46]. When 3 mg per 0.3 mL of α-glucosyl hesperidin was given three times a week for 31 days to the collagen-induced arthritis (CIA) rat model, it showed anti-RA effects through downregulating tumor necrosis factor (TNF) [47,48]. EGCG, a powerful compound from *Camellia sinensis*, had anti-RA properties for human rheumatoid joint pain aggravating synovial fibroblasts (RASF), when administered for 12 h, by downregulating epithelial neutrophil-starting peptide (ENA)-78, Regulated upon Activation, Normal T Cell Expressed and Presumably Secreted (RANTES), and growth-regulated oncogene (GRO)-IL-1-impelled MMP-2 [49]. In human RA synovial fibroblasts (RASFs), EGCG dosages of 125, 250, and 500 nM for 24 h inhibited the synthesis of MAPK, MMP-1, MMP-3, p-extracellular regulated kinases (ERK)1/2, p-JNK, p-p38, and AP-1 (RASFs) [50].

When CIA rats were treated with a 10 mg dose for each kg of body weight for three weeks, IL-6, TNF, and interferon (IFN)- were restrained, while anti-type II collagen (CII) explicit IgG1 antibodies were activated [51]. The restraint of myeloperoxidase by EGCG at ten mg/kg for five days uncovered the antagonist of RA benefits in pristane-induced arthritis (PIA), myeloperoxidase (MPO) [52], CTR, carbonic anhydrase II, cathepsins K, alpha- and beta-integrins, and NF-ATc1 all negatively responded in human osteoclasts and mice after 15 days of treatment at 20 and 50 M [53].

### 3.4. Other Compounds

Different polyphenols were examined similarly due to their contradictory properties against RA. Extra virgin olive oil (EVOO) polyphenol, which was extracted from extra virgin oil, inhibited RA in mice with collagen-induced arthritis (CIA). EVOO polyphenols were induced for about 2 weeks by downregulating TNF-α, IL-1, IL-6, pEG2, P38, JNK and P65 [54,55]. Another strategy for expanding CA bioavailability is by producing CM-stacked Ns (CM-Ns). They also used three different exploratory groups and one benchmark group to prepare for their trials. Nonetheless, there is no examination of the sub-atomic component of the CM-Ns antagonist of RA effects [56]. Another study examined how emodin influences the apoptotic pathway, concentrating on the B-cell lymphoma protein 2 (Bcl-2)-associated X (Bax) irregularity and caspase 3 and caspase 9 initiation [57].

Polyphenols play a leading role in reducing RA symptoms. However, oxidative, and apoptotic systems are not commonly discussed in the research. Polyphenols anti-RA properties have primarily been studied concerning inflammatory pathways. A few studies focused on polyphenols’ antioxidative and apoptotic effects, which reduce RA symptoms, but they were few. More exploration is expected to comprehend the atomic systems of polyphenols antioxidative and apoptotic activities in RA pathogenic pathways [58].

## 4. Plant Polyphenols Targeting Oxidative Stress and Inflammation

Polyphenols are metabolites developed by plants, including organic products, leaves, and bark. Polyphenols are abundant in numerous normal natural products (grapes, cherries, apples, pomegranates, and oranges) [59], spices, and flavors. Anti-inflammatory and antioxidant effects, as well as prevention agent impacts, are exerted by these substances. Polyphenols’ antioxidant properties depend on the capacity to scavenge ROS molecules, suppress prooxidant gene expression in articulation and advance the statement of antioxidant genes such as SODs and catalases [60,61].

They also manifest anti-inflammatory properties that are dependent on their capacity to inhibit pro-inflammatory signaling tracks including (MAPK), AP-1, and NF-κB. Various trials have shown that polyphenolic chemicals, mostly known for their antioxidant and anti-inflammatory characteristics, can help prevent OA [61,62,63]. Many polyphenols have been studied in vitro and in vivo model of OA, including pomegranate extracts, Butein, green tea polyphenol, EGCG, resveratrol, wogonin, quercetin, harpagoside, curcumin, morin, etc. It was recently showed that Butein, a chalcone-rich concentrate of *Butea monosperma* blossoms, just like unadulterated Butein, had significant cancer-preventing properties and repressed the creation of IL-six and metalloproteases in chondrocytes by expanding autophagy through the activated protein kinase (AMPK)/mTOR signaling pathway [62]. Butein enacts AMPK through expanding the phosphorylation of AMPKThr172 and hinders mTOR movement by reducing the phosphorylation of MTORSer-2448 [63].

Additionally, it was found that consolidating an extract of *Scutellaria baicalensis* with pure wogonins restrains the outflow of IL-6, COX-2, iNOS, and metalloproteases set off by IL-1, as well as the development of PGE2 and NO. In essential human chondrocytes, wogonins help the movement of Nrf2, the expert record controller of antioxidant defense proteins, as the creation of HO1 offers protection from IL-1-induced oxidative stress [64]. Harpagoside, an iridoid, repressed IL-1, induced the formation of MMP-13 and a large number of pro-inflammatory cytokines and chemokines, including IL-6, in essential human OA chondrocytes by restraining the cFos/AP-1 signaling pathway, which was free of the c-Jun and NF-κB pathways [65]. Harpagoside, when combined with glucosamine hydrochloride, chondroitin sulfate, methyl sulfonyl methane, and bromelain extracts, suppressed the production of IL-1 and TNF-α in a formalin-induced rat OA model [66].

Curcumin, a phenylpropanoid and the principal element of turmeric, has a pleasant flavor, with anti-inflammatory characteristics that have been illustrated. Curcumin and resveratrol are some of the most famous compounds known for their anti-inflammatory and medicinal properties, also showing various signaling molecule targets performing at the cellular level which supports OA and RA pathogeneses. TNF-α is the main regulator in OA and RA and this effect is maintained by the activation of NF-κB. Although, TNF-α is known to be the main potent NF-κB activator [67,68,69]. Curcumin’s chondroprotective movement has been indicated in various in vitro and in vivo examinations utilizing chondrocytes, cartilage explants, and an assortment of animal models [70,71,72]. The oral administration of curcumin and tetrahydro-curcumin reduced the production of IL-1, IL-six, and metalloprotease in rat and mouse models of experimental OA, while also relieving pain and cartilage degeneration [71]. Enzymatically altered curcumin decreased inflammation and retarded the course of OA in an anterior cruciate ligament transection (ACLT)-induced OA in rabbits model [73]. Ferulic acid (FA) [74], a curcumin derivative found in the cell dividers of different plants, which includes oats, rice, and orange and apple seeds, has anti-inflammatory and antioxidant characteristics, and has been shown to inhibit TNF and IL-1 expression when exposed to H_2_O_2_ [75]. Resveratrol has been shown to alleviate disease properties [76].

Resveratrol (trans-3,4′,5-trihydroxystilbene) is found mainly in grape skin and wine, peanuts, pistachios, blueberries, mulberries, cocoa and chocolate, soy, etc. The expression of iNOS and NO in Artesunate attenuates (ACLT) OA bunnies was diminished by intra-articular resveratrol infusions [77]. In rodents with experimental OA, resveratrol decreased the expression of IL-1, TNF-α, and IL6. Resveratrol repressed the NF-κB and AP1 signaling pathways [78], which decreased the AGE-instigated creation of iNOS, COX-2, and MMP-13 in chondrocytes [79]. Resveratrol enacted SIRT1 in chondrocytes, repressed NF-κB initiation, and diminished IL-1-activated iNOS creation in human chondrocytes [80]. Olive oil is high in polyphenols and is regularly consumed in the Mediterranean diet [81,82]. Olive oil has been shown to work on joint wellbeing and capacity in a few in vitro and in vivo examinations. The polyphenol hydroxytyrosol in olive oil activates autophagy and stops chondrocyte mortality [83]. In a rodent model of ACLT-prompted OA, the oral admission of a virgin olive oil-rich eating regimen had mitigating impacts, diminished IL-6 articulation, and expanded lubricin articulation [84,85]. The present study and other research back up the use of an olive oil-rich diet as a viable option for maintaining healthy joint function [83,84,85].

In addition to the plant-extracted compounds discussed above, a few extra polyphenols have been shown to lessen oxidative pressure and aggravation in chondrocytes, just as in OA pathogenesis. Imperatorin (a secondary metabolite found in *Apiaceae* and *Rutaceae* family plants) was shown to restrict iNOS articulation and NO age by restraining the ERK1/2-AP1(cFos/cJun) pathway [86]; it attaches to iNOS and inhibits its action, according to research findings [87].

Genistein, an isoflavone, decreased COX-2, iNOS, and NO generation in chondrocytes after exposure to LPS and IL-1 in an in vitro investigation. In monosodium iodoacetate (MIA)-induced OA in rats, an aqueous extract of Java tea (*Orthsiphonstamineus*) decreased inflammation and reduced the seriousness of OA in cartilage explants [88,89].

Olive and grape seed extracts high in hydroxytyrosol and procyanidins (HT/PCy) inhibited the production of iNOS, COX-2, and metalloproteases in chondrocytes triggered with IL-1 and displayed chondroprotective effects in post-traumatic OA models in mice and rabbits [90]. In vivo research showed that an oleuropein-enriched diet effectively reduced synovial inflammation and the blood levels of PGE2 in a guinea pig model of spontaneous OA. In human chondrocytes, chlorogenic acid therapy decreased the synthesis of PGE2 and NO and inhibited the expression of iNOS and COX-2 produced by IL-1 [91].

Therefore, polyphenols were found to scavenge ROS, activate the antioxidant defense system in chondrocytes, and block pro-inflammatory signaling pathways, which decreased inflammation. Future research should focus on delivering therapeutic levels of polyphenolic chemicals to the damaged joints, which is a fundamental restriction of OA treatment. This would improve the medication efficacy and joint health and function. In conclusion, polyphenolic compounds have the potential to develop as effective treatments for OA, according to recent studies.

### 4.1. Polyphenol’s Antioxidant Characteristics

Extreme ROS generation can cause tissue damage, which can trigger an inflammatory reaction. The chemical structures of polyphenol impact their cell antioxidant actions [92]. The amount of hydroxyl bunches fundamentally affects various cell antioxidant systems, including revolutionary searching and metal particle chelation abilities [93]. Polyphenol’s antioxidant movement is connected to its ability to search a broad scope of ROS. Suppressing ROS blend by repressing compounds engaged in their generation, searching for ROS, upregulating, or ensuring cell antioxidant guards are, overall, components engaged in the activity of polyphenols agent in cancer prevention [94].

Polyphenols might repress the synergistic action of compounds associated with the generation of ROS. Polyphenols protect against oxidative harm by producing hydrogen peroxide (H_2_O_2_), which helps in maintaining the immune responses such as cell growth [95]. By diminishing hydrogen peroxidase and producing responsive hydroxyl radicals, ROS has been displayed to build free metal particles. As a result of their ability to chelate metal particles (iron, copper, etc.) and free radicals, polyphenols with lower redox potential can thermodynamically lessen exceptionally oxidizing free revolutionaries. Quercetin, for instance, has iron-chelating and iron-balancing actions [96].

### 4.2. Polyphenols and Their Interactions with Free Radicals

Polyphenols might react with non-polar atoms in the hydrophobic internal layer of the plasma membrane, causing changes in the oxidation pace of lipids and proteins. A few flavonoids found in the layer hydrophobic center might help in keeping oxidants out and protect the layer uprightness and capacity. These cycles might also be considered in the understanding of polyphenols essential systems of activity, such as cell attachment and signal transduction [97].

The connection of polyphenols with the movement of nitric oxide synthases (NOS) may control NO production. A few flavonoids, for example, quercetin, stilbenes, and luteolin, were shown to smother the action of xanthine oxidase (XO), a basic generator of free radicals. Flavonoids may likewise forestall the emergence of these free radicals by neutrophils and the enactment of these cells by 1-antitrypsin, just as they decrease the movement of peroxidase [98].

### 4.3. Enzyme Inhibition Included in Oxidation

Different polyphenols manage the action of arachidonic enzymes (such as cyclooxygenase (COX), lipoxygenase (LOX), and NOS), by the creation of prostaglandins, leukotrienes, and NO, which mostly act as messengers and aggravation, and are reduced when these chemicals are hindered through the inflammatory arachidonic corrosive pathway [99].

Bacteria endotoxins and inflammatory cytokines might invigorate macrophages, prompting expanded iNOS articulation and NO age, as well as oxidative harm. Polyphenols might diminish oxidative harm by restraining (LPS)-initiated iNOS quality articulation and related movement in refined macrophages [100].

COX and LOX are enzymatic cycles that produce metabolites that can enhance oxidative injuries in tissues. Some polyphenols can stifle COX and LOX action. Metabolites, especially those produced in the XO course, may worsen tissue oxidative harm [101]. During ischemia, xanthine dehydrogenase (XDH) movement might change over to XO action, driving the development of ROS. Polyphenols were proved to decrease the action of XO by way of bringing down oxidative harm [102].

## 5. Anti-Inflammatory Polyphenols

### 5.1. Polyphenols Have Modulatory Effects on the Cells Involved in Inflammations

Dietary polyphenols are adjuvants that can improve the global management of RA due to their immunomodulatory and anti-inflammatory effects. Scientific evidence indicates that polyphenols interfere with the metabolic activity of dendritic cells, interact with macrophages, promote B cell and T cell proliferation and suppress Type 1 helper (Th1), Th2, Th9 and Th17 cells. Moreover, they are effective in both the adaptive and innate systems, involving stimulatory and inhibitory effects, depending on the interactions with the components of the immune system [103].

RSV may alter the differentiation of human dendritic cells from monocytes. This statement is supported by a study whose aim was to assess the regulatory actions of polyphenols [104]. EGCG also has an immunosuppressive effect due to its downregulation mechanisms acting on CD11c, CD80, CD83 and major histocompatibility complex (MHC) class II, which are required for antigen presentation by dendritic cells [105]. Furthermore, the inhibitory effects of polyphenols have also been demonstrated in a preclinical study using a mouse model that fisetin (50 mg/kg) inhibited the migration and allo-stimulatory capacity of dendritic cells [106].

Macrophages are classified into two groups based on their polarization: inflammatory M1 and immunosuppressive M2 phenotypes. The effect of cocoa polyphenolic bioactive molecule extract (caffein, quinic acid, chrysophanol-hexoside, vanillic acid derivative, catechin-3-*O*-glucoside, theobromine, cinnamic acid derivative, procyanidin B dimer, clovamide) on macrophage polarization has been evaluated in an experimental study and a phenotypic switch from the M1 to the alternative M2 state has been reported [107].

Natural killer (NK) cells have a potent cytolytic activity and a significant role in immunological processes. Perforin and granzyme B are cytoplasmic granule-associated proteins secreted by activated NK cells, which cause apoptosis and necrosis in target cells. Green tea catechin metabolites have immunomodulatory effects by promoting NK cell cytotoxicity through an increase in their activity [103].

The adaptive immune system’s main components are B and T cells, and the medical data suggest the involvement of polyphenols in the modulation of these cells. An experimental study conducted in vitro has reported that catechins may suppress the B cell production of immunoglobulin E (IgE), without being associated with cell necrosis and apoptosis [108]. Moreover, the immunoregulatory effects of polyphenols have also been determined in another experimental study which has shown their potential to inhibit the mitogen-induced proliferation of T cells and polyclonal immunoglobulin production by B cells, depending on the dose administered [109].

Treg cells are a type of T cell with a significant role in modulating auto-immunity processes. Medical evidence obtained from a preclinical study suggested that EGCG can stimulate Foxp3 expression, following the activation of Treg and the suppression of cytotoxic T cell function [110]. Furthermore, it has been reported in preclinical studies that RSV reduces the quantity of Th17 cells and pro-anthocyanidins modulate the Th17/Treg ratio [103].

In animal models of intense and ongoing aggravation, polyphenols such as quercetin, rutin, morin, hesperetin, and hesperidin were shown to have mitigating properties [96]. Rutin is helpful in constant provocative illnesses, such as joint pain, while flavanones are additionally viable in xylene-instigated neurogenic irritation. Carrageenan-actuated paw edema has been demonstrated to be decreased by quercetin. Daidzin, glycitin, genistein, and their glucosides can adjust the intense reaction produced by LPS infusion [111].

Polyphenols can possibly impact provocative enzymatic and signaling systems, for example, tyrosines and serine-threonine protein kinase. The following catalysts are perceived to play a part in cell enactment, immune system microorganism multiplication, B lymphocyte activation [112] and others, or the generation of cytokines by activated monocytes. Genistein has been distinguished as a tyrosine-protein-specific inhibitor kinase [113,114]. T cell augmentation is followed by the phosphorylation of tyrosine of specific proteins; this last substance could be responsible for a part of the calming effect. Polyphenols additionally affect inflammatory cells secretory cycles. Luteolin, kaempferol, apigenin, and quercetin have been shown to be effective inhibitors of the chemicals β-glucuronidase and lysozyme delivered by neutrophils. Moreover, these polyphenols substantially reduce arachidonic corrosive delivery from cell layers [115].

The results of preclinical studies are promising, but further research is needed in order to extrapolate the results to RA patients.

### 5.2. Mechanism of Anti-Inflammatory Effects of Polyphenols

Polyphenols might have anti-inflammatory impacts, particularly through radical scavenging, cell movement guidelines in inflammatory cells, and action regulations in digestion with arachidonic corrosive, arginine digestion (phospholipase A2, COX) (NOS), and the modulation of other generations of proinflammatory atoms. The hindrance of pro-inflammatory provocative catalysts, such as COX-2, LOX, and INOS, hinders NF-κB and actuates protein-1 (AP-1), enacting stage II cancer prevention agent detoxified chemicals and initiating (MAPK), protein kinase-C, and nuclear factor erythroid 2-related subatomic components for polyphenol-mitigating activities [113]. Solid proof comes from a normal phytochemical analysis that exhibited the adjustment of various inflammatory mediators, for example, arachidonic corrosives, various peptides, excitatory amino corrosive cytokines, and acids, determining metabolites. Likewise, in other exercise messaging (cGMP, cAMP, protein kinases, and calcium), certain record components, chemicals, and mixtures (AP-1, NF-fraction, and protocol conservations), (iNOS, COX-2), neuropeptides, proteases, and cytokines (IL-1β, TNF-α), in the aggravation interaction, are known to be focal [116].

An experimental study assessing pomegranate peel polyphenolic compounds has reported that TNF-α, IL-1β, IL-6 pro-inflammatory cytokines, NO and PGE2 inflammatory mediators were found to be at lower levels, due to the actions of punicalagin (PA) and ellagic acid (EA) on iNOS and COX-2 expression [117]. Moreover, it has been reported that PA and EA also inhibit the LPS-induced production of ROS and suppress TLR4, a protein with significant roles in inflammation [103].

The anti-inflammatory mechanisms of polyphenols can have an impact on RA management and are based on interactions with different signaling pathways, generating different immune responses, as follows:

- Cucurmin suppresses NF-κB, reduces IL-1β and stimulates IL-6 and vascular endothelial growth factor (VEGF) by rheumatoid arthritis fibroblast-like synoviocytes (RA-FLS);
- Cucurmin stimulates IL-6 and VEGF by RA-FLS and induces the apoptosis of RA-FLS;
- RSV inhibits Th-17, B-cells and the MAPK signaling pathway and reduces IL-6 and IL-1;
- EGCG suppresses NF-κB and MAPK and inhibits osteoclast differentiation;
- Extra virgin olive oil polyphenol extract (oleocanthal, oleacein, ligstroside aglycone monoaldehyde) reduces TNF-α, IL-1β, IL-6 pro-inflammatory cytokines, COX-1 and NF-κB translocation [53];
- Quercetin alters phosphatidylinositol 3-kinase/protein kinase B signaling pathway and reduces IL-1 and IL-6 [103].

Medical records suggests that polyphenols may help RA patients enhance their quality of life.

## 6. The Roles of Polyphenols in MAPK Pathway in Rheumatoid Arthritis

Polyphenols are the plant secondary metabolites that can release a signaling cascade, which can be neutral or detrimental to cell survival. Toll like-receptor (TLR) is the class of pattern recognition receptors (PRRs) which plays a major role in innate immune responses. TLR activation triggers several different pathways. The key signaling proteins are mitogen-activated protein kinases (MAPKs), key pathways in the development of RA. In a physiologically normal state, MAPK loops are important signaling pathways that play a role in a variety of processes in the control of cell proliferation, survival, and differentiation of healthy cells. Nonetheless, TLR-dependent MAPK pathway activation mediates pro-inflammatory cytokine expression in macrophages and RA synovial fibroblasts (SF), which promotes joint damage and persistent inflammation. MAPKs are serine/threonine protein kinases that are extremely conserved [118,119].

Extracellular stimuli such as cytokines, TLRs, neurotransmitters, and oxidative stress primarily activate them. They stimulate corresponding receptors, which then transduce intracellular signaling into the nucleus via three primary MAPK cascades. In humans, the three primary kinase chains are extracellular signal-regulated kinase (ERK) 1/2, C-jun N-terminal kinase (JNK), and p38 MAPK [120]. Intracellular kinases (such as MAPKK, MEK, or MKK, as well as MAPK) are hypothesized to initiate the downstream activation of MAPKs (ERK1/2, JNK, and p38 MAPK) by phosphorylating serine, threonine, or tyrosine residues in the appropriate protein [121,122,123]. The dynamic MAPKs (ERK1/2, JNK, and p38 MAPK) phosphorylate the proper record characteristics and move into nuclei, where they impact quality gene expression [124].

The MAPK signaling pathway has been observed to be dynamic and associated with the pathophysiology of RA. In the RASFs, there is a raised measure of phosphorylated p38 MAPK. Additionally, upgraded ERK and JNK signaling particle expression has been found in RASFs and the macrophages of RA patients [125].

Previous research has uncovered that actuating the TLR-dependent MAPK signaling pathway causes transforming growth factor beta (TGF-β), VEGF, HIF-1, and MMPs to be initiated in RASFs, bringing about RASF multiplication and synovial hyperplasia. The MAPK signaling pathway in RA builds the statement of key favorable to an incendiary go-between, development components, and MMPs by stimulating RASFs and synovial macrophages with the support of fiery cytokines like TNF-α, IL-1, and IL-6. In RA, the effect of the P38 MAPK pathway on persistent aggravation and the creation of pro-inflammatory cytokines is being examined [126]. According to research, many pro-inflammatory cytokines in RA are thought to be mediated by the p38 MAPK pathway. In numerous RA disease models, the specific inhibition of p38 MAPK has been demonstrated to reduce joint deterioration and TNF-α release. There are four isoforms of p38 that have been identified so far. In RA, the p38 isoform plays a key role in the generation of inflammatory cytokines by synovial macrophages. JNK is another key MAPK signaling molecule involved in the creation of MMPs in RASFs and synovial macrophages. Conversely, a JNK inhibitor study found that restraining the JNK-intervened enactment of AP-1, collagenase-3, and MMP articulation shielded rodents from bone weakening in an adjuvant-induced ligament rat model. In inclusion, the principal work of other upstream MAP kinase kinases (MAPKKs) like MEKK-2, MKK-4, and MKK-7 in RA pathogenesis has also been mentioned [127].

### Epigallocatechin-3-Gallate, Magnolol, and Other Polyphenols’ Anti-Inflammatory Properties against RA, via MAPK Pathway

Bioactive compounds have been identified as key mediators in the pathogenesis of RA, and they may lead to a prospective treatment goal. Multiple investigations have revealed JNK, a crucial factor in joint deterioration in inflammatory arthritis, to be widely recognized [128].

More bioactive substances have been identified in RA for their efficacy in reducing disease severity, mostly via regulating the TLR/MAPK signaling system. Many studies have recently been published that show that bioactive substances may play a function in TLR-mediated MAPK signaling pathways. In LPS-induced RAW 264.7 cells, bioactive substances such as tanshinone IIA and alternaramide were found to inhibit NF-κB, MAPK, and TLR-4 MYD88-mediated pathways [129,130].

Furthermore, there are studies which demonstrated that anti-arthritic natural pyrano-chalcone-derived compounds downregulated LPS-induced NF-κB, TLR-4, JNK, and ERK expression in a collagen-induced arthritic (CIA) rat model [131], as indicated by in vivo experiments. The green tea polyphenol EGCG has been demonstrated to reduce IL-12 production and alleviate RA and some other inflammatory illnesses by inhibiting ERK and p38 MAPK activation. In RA synovial fibroblasts, EGCG therapy reduced the TNF-induced phosphorylation of all three major classes of MAPKs, including ERK, p38 MAPK, and JNK. Curcumin, the key active ingredient in turmeric, has been shown to suppress ERK1/2 and p38 while activating JNK, c-Fos, and NFATc-1 in RA patients’ peripheral blood mononuclear cells (PBMCs), a considerable reduction in pro-inflammatory cytokines [132].

Additionally, in vitro studies have indicated that RA patients show less phosphorylation of MAPK signaling molecules, which inhibits osteoporosis and bone degradation [133]. Furthermore, they demonstrated that phloretin can block the NF-κB and MAPK pathways, potentially limiting T cell activation and macrophage-mediated inflammatory cycles. Anti-arthritic effects of pomegranate-derived polyphenol, particularly punicalagin (PA) and ellagic acid (EA), have been found to reduce cartilage degradation by inhibiting IL-1-induced p38-MAPK activation in human osteoarthritis chondrocytes [134].

The pure polysaccharide ESP-B4, which is a key component of *Ephedra sinica* acidic polysaccharides, has recently been shown to have an immunosuppressive effect on RA. Eosinophil stimulation promoter (ESP)-leukotriene B4 inhibited the TLR-4 signaling tracks and the phosphorylated MAPKs caused by LPS stimulation in in vitro and in vivo experiments, reducing the production of inflammatory cytokines and mediators [135].

Magnolol, a naturally occurring phenolic molecule, has been shown to have anti-inflammatory properties in RA patients by inhibiting lipopolysaccharide receptors’ LPS-induced TLR-4 expression, TLR-4-mediated MAPK signaling, and the production of pro-inflammatory cytokines [136]. However, the exact mechanism of *Tripterygium wilfordii* hook factor (TwHF)-mediated miR-146a regulation and the bioactive molecule found in TwHF have yet to be discovered. The importance of bioactive substances in the control of microRNAs in RA is highlighted in this context [137,138]. As a result, targeted microRNA balancing via prospective bioactive medicines is a viable technique in TLR/MAPK signaling and RA reduction [139].

**Figure 4:**
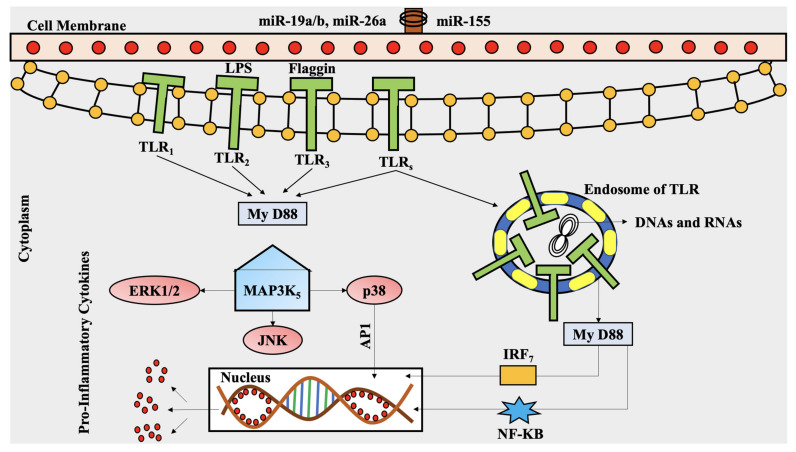
The involvement of numerous bioactive substances in the TLR-dependent MAPK signaling cascade in RA. The TLR receptor attracts (MyD) 88 and other related adaptor proteins are activated (ERK1/2, JNK, P38). The MyD88-dependent system controls the expression of pro-inflammatory cytokines and other immune-related genes, signaling through the MAPK pathway, which in turn leads to the activation of the transcription factor AP-1 in RA. ERK: extracellular regulated kinase; IRF: interferon regulatory transcription factor; My D88: myeloid differentiation primary response 88; TLRs: Toll-like receptors; LPSs: lipopolysaccharide receptor; MAPK: mitogen-activated protein kinase; JNK: Jun N-terminal kinases; NF-κB: nuclear factor kappa-light-chain-enhancer of activated B cells; miR: microRNA; AP1: activator protein 1; p38: a MAPK.

The involvement of numerous bioactive substances in the TLR-dependent MAPK signaling cascade in RA is depicted in Figure 4.

## 7. p53 Gene Mutation via Oxidizing Agents in RA

The persistent inflammation of RA has been predicted to cause DNA damage serious enough to determine p53 changes and different transformations in the cell cycle and growth suppressant qualities [140]. Surely, in patients with RA synovial tissue, the measure of fractured DNA is essentially higher than with controls [141]. Additionally, the high p53 articulation transformations may help to explain FLS and insufficient apoptosis changes in aggregate observed in rheumatoid synovial tissue [142].

Refined FLS and RA synovial tissue cDNA (complementary DNA) were analyzed with RNA location to determine the contribution of p53 in RA; p53 transformations in RA were found. Following subclone and subsequent series examinations, around 40% of the p53 cDNA was shown. Clones containing mutations are isolated from the rheumatoid synovium [141].

This is thought to explain why single-stranded conformation polymorphism (SSCP) testing or standard sequencing is not sensitive enough to detect rheumatoid synovium changes [143]. Transformations normal for oxidative deamination were changed in >80% of the cases available. The presence of p53 transformations in the condition and FLS culture from long-haul erosive patients has recently been affirmed, albeit the outcomes differ. A new report has shown p53 FLS changes in the synovium of RA American patients [144].

Transformations of p53 can likewise help in the overproduction in the rheumatoid synovium of cytokines and metalloproteinases. Mutant p53 protein fails to suppress interleukin 6 and metalloproteinase 1, encoding qualities for the ex-accessibility of the p53 protein. Moreover, the yield of NO might keep on developing. TNF-α and insulin-like factor necrotization may impact the appearance of p53, a factor of development that can play a role in downregulation and upregulation in p53 [145,146]. Table 1 summarizes the polyphenols inhibiting RA.

## 8. Future Directions and Conclusions

Cytokine release, angiogenesis, osteoclast activity and oxidative stress lead to inflammatory processes in synovial joint tissue and have all been linked to the progression and severity of RA, making them ideal targets in the research for therapeutic improvement [3,4,43,45,48]. The literature investigated in this review shows that polyphenolic compounds (such as EGCG, butein, wogonins, resveratrol, curcumins, etc.) have very effective anti-inflammatory properties, being also cancer prevention agents.

Disease-modifying anti-rheumatic drugs and surgical procedures have been unable to fully control the onset and outcome of RA, so there is a critical need to develop innovative and safe compounds as an alternative to the current management of this disease. Polyphenolic compounds have a lot of potential to become a priority choice in order to control oxidative damage. The results of numerous studies conducted with animal and cell models have shown the potential effectiveness of polyphenols as adjuvant treatments in the global management of RA. However, only a few clinical trials involving a small number of patients have been conducted to determine the possibility of extrapolating the results to human beings, so further research is needed in order to assess their efficacy and safety profiles [148].

Anti-inflammatory supplements and diets consisting of foods rich in phenolic compounds may be a way to emphasize prevention over therapy. Exogenous antioxidants are increasingly important in order to manage the oxidative damage specific to RA. Moreover, the results of medical studies conducted on polyphenols may represent a starting point in the development of chemopreventive compounds with favorable safety and efficacy profiles [149].

The absorption of polyphenols is limited, and ingested polyphenolic compounds are intensively metabolized by phase II reactions. Pharmaceutical development should also focus in the future on the synthesis of derivates with a higher bioavailability [150].

## 9. Conclusions

In this review, an overview was presented of the implications of polyphenolic compounds in the MAPK pathway in RA. In recent years, the importance of polyphenols in mitigating RA due to their antioxidative and anti-inflammatory effects has been more and more recognized, making them promising tools for RA adjuvant therapy as new pathophysiological mechanisms of RA are being discovered.

Polyphenols alleviate the symptoms of RA by modulating a wide range of RA-related molecules, including MAPK, ILs 1 and 6, TNF-α, NF-κB, JNK, ERK1/2, AP-one, and COX-2. Polyphenol’s anti-RA efficacy has primarily been studied in terms of its impact on inflammatory pathways. The mechanistic explanation of polyphenol antioxidative, anti-inflammatory, and apoptotic activities, which also control RA pathogenic systems, needs more research. Clinical studies could be carried out based on the preclinical data. The specific exchange of combinational miRNAs associated with negative regulation of TLR/MAPK enactment across many tissues or cell types could be an effective therapeutic method for the future treatment of RA. Based on miRNA research, new potential biomarkers and innovative diagnostic methods are expected to be developed in the near future. A better understanding and description of the systems that are thought to require polyphenols in adverse situations would aid in medically clarifying those situations where polyphenol consumption will be beneficial. In addition, such research could aid in the creation of new anti-inflammatory drugs. These polyphenols have been displayed to scavenge ROS; furthermore, they initiate the antioxidant limitation system in chondrocytes, and suppress inflammation by hindering supportive pro-inflammatory signaling pathways.

Despite the antioxidant, anti-inflammatory and immunomodulant properties of polyphenols, there are no dietary recommendations for RA patients. There are many polyphenolic compounds, and their chemical structures influence their biological activities, including specific interactions with protein receptors. Therefore, it is important to perform qualitative and quantitative analyses on polyphenols from different extracts.

Larger clinical trials and a better understanding of the mechanisms underlying the inflammatory processes in RA and the pharmacological effects of polyphenols may help to define more precisely and to validate the clinical conditions in which the administration of polyphenols can be useful.

## Figures and Tables

**Table 1:** Rheumatoid arthritis-inhibiting phenolic acid, stilbenes, flavonoids, and other polyphenols.

Polyphenols	Source	Experimental Setup	Actioning Mechanism	Ref.
Ferulic acid	apple, sugar beet, popcorn, grains, vegetables	Macrophage, monocytes, Rats	NFATc1, c-Fos, NF-κB, MMP	[32]
*N*-feruloyl serotonin	safflower seed	AA	CRP, LOX, TNF-α, iNOS, IL-1β	[33]
Resveratrol	red grapes, peanut, soy	CIA, FLS	COX-2, PGE2, NADPH oxidase, ROS, p38, MAPK, ERK1/2, NF-κB	[39]
Epigallocatechin-3-gallate	green tea, strawberries, blackberries	CIA rat	IL-6, TNF-α, IFN-γ	[129,130]
Gallic acid	cinnamon bark	AIA rat	TNF-α	[32,33,147]
EVOO polyphenol extract	EVOO, fruit of olea, olives	CIA	TNF-α, IL-1β, IL-6, PEG2, p38, JNK, p65	[55]
Curcumin	turmeric rhizome	RA-FLS	IL-1β, IL-6, NF-κB, ERK1/2	[70]
*p*-Coumaric acid	gnetum	AIA	TNF-α, IgG	[34]
Emodin	rhubarb, asian knotweed	Synovial membrane in human	MMP-1, MMP-9, NF-κB, MAPK	[57]
Hesperidin	soybean, sweet orange, tangerine	Wistar rat	GSH, SOD, catalase	[47,48]

AA: amyloidosis; AIA: adjuvant-induced arthritis; NFATc1: nuclear factor of activated T cells cytoplasmic-1, NF-κB: nuclear factor kappa light chain enhancer of activated B cells, MMP: matrix metalloproteinases, COX-2: cyclooxygenase, PGE2: prostaglandins E2, NADPH: nicotinamide adenine dinucleotide phosphates, ROS: reactive oxygen species, MAPK: mitogen-activated protein kinase, ERK1/2: extracellular signal-directed kinase, IL: interleukin, TNF: tumor necrosis factor, IFN-γ: interferon gamma, PEG2: polyethylene glycol-2, p38: phospho-p38 mitogen-activated protein kinase JNK: Jun N-terminal kinases, p65: phospho-p65 mitogen-activated protein kinase, IgG: immunoglobulin G, EVOO: extra virgin olive oil, CIA: collagen-induced arthritis, AIA: rat adjuvant arthritis, RA-FLS: rheumatoid arthritis-fibroblast-like synoviocyte; GSH: glutathione; SOD: superoxide dismutase.

## Abbreviations

LOX	12-15 lipoxygenases
AMPK	Activated protein kinase
AP1	Activator protein 1
AP-1	Activator protein-1
ACLT	Anterior cruciate ligament transection
ACPA	Anti-citrullinated protein antibodies
ACLT	Artesunate attenuates
BCL-2/Bax	B-cell lymphoma protein 2-associated X
BMMs	Bone marrow macrophages
JNK	c-Jun N-terminal kinases
CRP	C-reactive proteins
CAT	Catalase
CIA	Collagen induced arthritis
COX	Cyclooxygenase
DLN	Draining lymph node
EA	Ellagic acid
ESP-B4	Eosinophil stimulation promoter -leukotriene B4
EGCG	Epigallocatechin-3-gallate
ESR	Erythrocytes sedimentations rate
EVOO	Extra virgin olive oil
ERK	Extracellular signal-directed kinase
FA	Ferulic acid
FLSs	Fibroblast-like synoviocytes
GSH	Glutathione
GPx	Glutathione peroxidase
GR	Glutathione reductase
GRO	Growth-regulated oncogene
HLA	Human leukocyte antigen
H_2_O_2_	Hydrogen peroxide
HO2	Hydroxyl radical
HT/PCy	Hydroxytyrosol and procyanidins
IgG1, IgG2a	Immunoglobulins G
IFN	Interferon
IL	Interleukin
LPS	lipopolysaccharide
MnSOD	Manganese-subordinates superoxide dismutase
MAPKKs	MAP kinase kinases
MMP	Matrix metalloproteinases
MAPK	Mitogen-activating protein kinase
MIA	Monosodium iodoacetate
MPO	Myeloperoxidase
NADPH	Nicotinamides adenine dinucleotide phosphates
NO	Nitric oxide
NO_2_	Nitrogen dioxide
NF-κB	Nuclear factor kappa light chain enhancer of activated B cells
NFATC1	Nuclear factor of activated T cells cytoplasmic-1
OA	Osteoarthritis
OPG	Osteoprotegerin
p38	a mitogen-activating protein kinase
PRRs	Pattern recognition receptor
OONO	Peroxynitrite
PIA	Pristane-induced arthritis
PGE2	Prostaglandins E2
PA	Punicalagin
AA	Reactive amyloids
RNS	Reactive Nitrogen Species
ROS	Reactive oxygen species
RANKL	Receptor activator of nuclear factors kappa-B-ligand
RANTES	Regulated upon Activation, Normal T Cell Expressed and Presumably Secreted
RSV	Resveratrol
RA	Rheumatoid arthritis
RA-FLS	Rheumatoid arthritis fibroblast-like synoviocytes
RF	Rheumatoid factor
RASF	Rheumatoid pain aggravation synovial fibroblasts
SSCP	Single-stranded conformation polymorphism
SOD	Superoxide dismutase
SJC-28	Swollen 28-joint count
SF	Synovial fibroblast
TJC-28	Tender 28-joint Count
TR	Thioredoxin reductase
TLR	Toll-like receptor
TGF-β	transforming growth factor beta
TwHF	Tripterygium wilfordii hook factor
TNF-α	Tumor necrosis factor
CII	Type II collagen
UV	Ultraviolet
UCOC	Uncarboxylated osteocalcin
VEGF	Vascular endothelial growth factor
XDH	Xanthine dehydrogenase
XO	Xanthine oxidase
